# Hidradenitis Suppurativa Burdens on Mental Health: A Literature Review of Associated Psychiatric Disorders and Their Pathogenesis

**DOI:** 10.3390/life13010189

**Published:** 2023-01-09

**Authors:** Stefano Caccavale, Vittorio Tancredi, Maria Pia Boccellino, Graziella Babino, Elisabetta Fulgione, Giuseppe Argenziano

**Affiliations:** Dermatology Unit, Department of Mental and Physical Health and Preventive Medicine, University of Campania Luigi Vanvitelli, 80131 Naples, Italy

**Keywords:** hidradenitis suppurativa, psychiatric, depression, anxiety, schizophrenia, bipolar, suicide, drug abuse, inflammation

## Abstract

Skin, mental health and the central nervous system (CNS) are connected by a deep link. It is not only the aesthetic and sometimes the disfiguring aspects of dermatological conditions that can cause a severe psychological burden; also, different studies have shown how chronic skin-inflammatory diseases may influence the activity of the CNS and vice versa. Moreover, the skin and brain share a common embryogenic origin. Hidradenitis suppurativa (HS) is a chronic inflammatory skin disease affecting the hair follicles of the apocrine regions. The main clinical features are nodules, abscesses, cysts, fistulae and disfiguring scars. Pain and stinking discharge from fistulae are often present. It is not surprising that the psychological burden associated with HS is frequently a challenge in dermatologists’ daily routines. Patients often suffer from depression and anxiety, but also from substance abuse, psychotic and bipolar disorders and an increased suicide risk. The aim of this article is to review the main psychiatric disorders associated with HS and their pathophysiology. Research on Pubmed was conducted with the key words Hidradenitis suppurativa, psychiatric, depression, anxiety, bipolar, schizophrenia, abuse, suicidal. A high incidence of psychiatric disorders has been described in HS compared to controls. Hidradenitis suppurativa is not a rare disease, and acknowledging the HS psychological burden, psychiatric-associated diseases and associated biomolecular pathways will help dermatologists to better care for their patients.

## 1. Introduction

Hidradenitis suppurativa (HS), or acne inversa, is a chronic inflammatory skin disease that affects about 1–4% of the population, but the prevalence is supposed to be higher due to often-delayed diagnosis. Hidradenitis suppurativa affects both males and females, but it is more common among women. The onset is usually after puberty [1,2]. The aetiology is unknown but could be multifactorial. A genetic component is involved. Some studies have shown that patients with HS may carry mutations in genes encoding the γ-secretase complex: nicastrin (NCSTN), presenilin 1 (PSEN1), presenilin enhancer 2 (PSENEN) and POGLUT1, an O-glucosyltransferase involved in the Notch pathway. PSEN1 mutations have been shown to mediate the cytokine and chemokine expression in macrophages, prolonging the inflammatory process that enhances HS [3,4].

**Figure 1:**
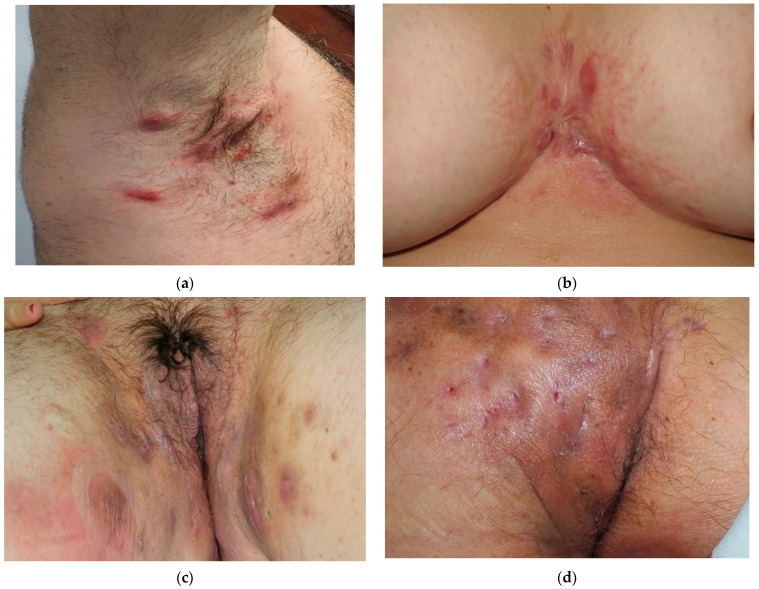
Clinical images of patients with hidradenitis suppurativa: nodules, scars and fistulae are detected in apocrine regions: axilla (**a**), intermammary sulcus (**b**), genitalia (**c**) and buttocks (**d**).

Clinically, HS presents with erythematous and painful nodules, mostly localized at the axillae, groins, buttocks and breast region. The subsequent formation of fistulae and scarring can occur. The discharge of stinking purulent material is also frequent, although cultures from suppurative lesions are usually negative; in fact, its origin is inflammatory and not microbial. However, secondary infections are possible and can worsen the course of the disease [5] (Figure 1).

Primary alteration seems to be determined by a modification of the pilo-sebaceous unit and the consequent occlusion of the terminal follicular epithelium in the body’s apocrine regions. This leads to the rupture of the follicles and skin inflammation with the infiltration of neutrophil cells and lymphocytes. The pilo-sebaceous unit alteration seems to be confirmed by the presence of hair tracts in 80% of HS lesions at ultrasonographic examination [6].

Obesity and smoking have been shown to play a central role in the pathogenesis of the disease. Increased adipose tissue is associated with systemic low-grade inflammation. Visceral adipocytes, as endocrine cells, secrete pro-inflammatory cytokines and fat acids that can exercise a chemotactic inflammatory response leading to abscess formation and the subsequent destruction of the pilo-sebaceous unit and other adjacent structures. Moreover, in HS obese patients, skin fold friction, sweating and inflammation can exacerbate HS manifestations. [7]. Smoking is commonly observed in HS patients, and some authors hypothesize that nicotine can promote follicular plugging [8].

The most used staging system is the Hurley classification based on the presence of abscesses without fistulas or scars (stage I), abscesses with tracts and scarring (stage II) and multiple diffuse abscesses (stage III) [8]. Hidradenitis suppurativa is also possible to stage sonographically, providing anatomic information that is often clinically unavailable [9].

Hidradenitis suppurativa treatment is related to disease severity: mild HS forms are usually treated with topical steroids and antibiotics. Severe HS requires more advanced therapies. Systemic antibiotics (mostly clindamycine and rifampicine) and anti-Tumor Necrosis Factorα (TNFα) antibodies (mostly adalimumab) are the most used. Beyond monoclonal antibodies, new therapies include: apremilast, a phosphodiesterase-4 inhibitor and Janus kinase (JAK) signaling blockers such as tofacitinib and upadacitinib [10]. The non-pharmacological therapies of HS comprise minor procedures such as carbon dioxide (Co2) laser, Intense Pulsed Light (IPL), incision, drainage and deroofing and major surgical procedures such as wide radical excision and local reconstruction with skin flaps or grafts. Hidradenitis suppurativa surgery often requires wide excision with problematic wound closure. A new reconstructive technique is represented by the co-graft Acellular Dermal Matrix (an acellular matrix made by collagen) and the Split Thickness Skin Graft [11,12].

The pain and associated itch, the unpleasant smell coming from the purulent discharge and the comorbidities such as obesity and sleep disturbance often associated with HS, as well as the stigmatization the patients often experience as a result of their disease, have a profound impact on patients’ quality of life and their mental health status. There are numerous validated instruments in order to assess quality of life in these patients; among them, the dermatology-specific DLQI is the most widely used one. Among the therapeutic options available, surgery seems to be the most effective in improving the quality of life of HS patients [13,14]. The correlation between mental health, the central nervous system and skin has been widely explored. Not only the aesthetic and sometimes disfiguring aspects of dermatological conditions may have a severe psychological burden, but also different studies have shown how chronic skin inflammatory diseases may influence the activity of the central nervous system (CNS) and vice versa [15]. Moreover, the skin and brain share a common embryogenic origin. It is not surprising that the psychological burden associated with HS is frequently a challenge in daily dermatological routines. Patients can suffer from depression and anxiety, but also from substance abuse, psychotic and bipolar disorders and an increased suicide risk. The aim of this article is to review the main psychiatric disorders associated with HS and their pathophysiology.

## 2. Materials and Methods

Our manuscript is a narrative review. We researched the principal scientific databases such as Pubmed, Scopus and so on. Research was conducted with the key words “hidradenitis suppurativa” or “acne inversa”, and the main psychiatric diseases such as “depression”, “anxiety disorder”, “bipolar disorder”, “substance abuse” and “suicide”. Furthermore, we carried out secondary research with the combinations of key words: “hidradenitis suppurativa”, “acne inversa”, “psoriasis”, “atopic dermatitis” and words relevant to the pathogenesis of psychiatric comorbidities associated with chronic skin inflammatory diseases such as “neuroinflammation”, “IL-6”, “TNFα”, “IL-17”. Only English works were evaluated. Only articles with titles and abstracts pertinent to our review were included. From a total of 185 results, 40 articles were analyzed. No restriction of pertinent data was preliminarily performed, but in the cases of repetition of concepts or data, only more recent results were selected.

## 3. Results

### 3.1. Depression

Depression is a mental status associated with poor mental health and/or losing interest in daily life activities. It is a common condition; the epidemiology is variable with a higher prevalence in women. The main symptoms are sadness, irritability, anhedonia, sleep and food intake disturbances. Depression can be primary or secondary to other medical or psychological conditions. Different subtypes are described according to intensity, cyclicality and principal clinical manifestations. Suicide can affect about 15% of patients suffering from depression [16]. Due to its chronic, often painful course but also social stigma, HS is often associated with depression. Different studies in the literature analyze this issue. Senthilnathan, A. et al. recruited 153 patients suffering from HS [17]. A Patient Health Questionnaire (PHQ-9) was used to assess depression. They found that 33% of patients had a major depressive disorder. No significant relationship was found between HS severity and depression, so even an objectively mild HS may have the same psychological impact as a more severe form of the disease. A.J. Onderdijk et al. studied the prevalence of depression and poor quality of life in 211 HS patients compared to 233 patients with other skin diseases [18]. Depression was assessed with the Major Depression Inventory (MDI) questionnaire. The mean MDI results were significantly higher in patient with HS [11.0 vs. 7.2 (*p* < 0.0001)]. On the other hand, the prevalence of depression in HS was not significantly higher (9% vs. 6%, *p* = 0.34) if using the MDI score to rate depression according to the International Classification of Diseases, 10th edition (ICD-10) criteria. In a recent systematic review and meta-analysis, Machado et al. evaluated the prevalence of depression in HS, including 10 studies comprising 40 307 participants. They found that the overall prevalence of depression was 16.9% (95% Confident Interval (CI), 9.9–27.2%) [19]. It is interesting to observe that in studies that use clinical criteria for depression diagnosis the prevalence was lower, at 11.9% (95% CI, 4.9–26.2%), compared to studies that use a screening diagnostic tool (26.8%, 95% CI, 20.4–34.5%). Moreover, the odds ratio (OR) for depression in patients with HS compared to individuals without HS was 1.84 (95% CI, 1.57–2.15). A more recent systematic review and meta-analysis, including a total of 38 studies, evidenced that for every four HS patients, one had depression [20]. The prevalence of depression in HS groups versus non-HS was 26.5% vs. 6.6%. The OR was 2.54 (95% CI, 2.15–3.01). Jalenques et al. also reported similar results: including 37 articles, the estimated depression prevalence in HS was 21% (95% CI [17,18,19,20,21,22,23,24,25]) [21].

Even if the prevalence of depression in HS seems to be frequent, currently relatively few hypotheses have been formulated with regard to this association. From the literature, three main theories have arisen. Quality of life impairment in HS patients is largely established. In particular, HS impacts on social, sexual and working activities. Acute disease provokes pain, stinking pus discharge and discomfort, which cause suffering, embarrassment and unease. All these factors can lead to depression. Moreover, HS is associated with other comorbidities such as obesity, chronic metabolic diseases and so on. Some authors believe this can impact significantly on patients’ mental health. Finally, it has been studied in psoriasis and atopic dermatitis that inflammatory cytokines may influence the release, uptake and metabolism of neurotransmitters and their receptors. A similar mechanism is postulated to be present in HS, too.

### 3.2. Anxiety

Anxiety is a normal reaction to stressful and potentially harmful daily situations, characterized by fear and adaptive physical reactions. Usually, it is accompanied by signs and symptoms of adrenergic cascade such as palpitations, cold sweats, tremors and so on. Anxiety is considered pathological when there are no trigger factors or when the reaction is abnormally severe compared to the situation involved. It is more common in younger people and in women. Anxiety can be associated with depression and other psychiatric conditions, but usually not with psychotic disorders. In some patients, suicidal behavior is present [22]. Some studies analyzed the prevalence and the risk of developing anxiety in HS. In a systematic review and meta-analysis, a prevalence of 12% (95% CI [6,7,8,9,10,11,12,13,14,15,16,17]) with an OR of 1.97 (95% CI [1.65–2.35]) was found [21]. In another meta-analysis and systematic review, Patel et al. found that anxiety was present in 18% of patients with HS versus 7% of the non-HS population, while the risk of developing anxiety was twice that of the second population without HS (OR, 2.00; 95% CI, 1.66–2.42) [20]. In contrast, another systematic review and meta-analysis estimated that the prevalence of anxiety was 4.9% (95% CI, 1.7–13.2%), while it was not possible to determine the OR [19]. The data available about anxiety prevalence are extremely variable, probably depending on the different diagnostic criteria used. Studies that use subjective questionnaires usually overestimate the results. Anyway, even if the rates vary, studies agree that anxiety is common in HS. As with depression, the exact mechanism of this association is not known, but it is likely to be multifactorial. Psychosocial factors certainly play a role, as HS represents a huge burden on patients’ lives from family to work, school, daily routine, sexual life and so on. Some studies show how a chronic disease, such as a metabolic disorder, and thus also HS, can be a severe burden on mental health. In particular, chronic inflammation is associated with elevated levels of pro-inflammatory cytokines that in psoriasis have been shown to play a crucial role in the development of mental health problems [23].

### 3.3. Bipolar Disorder

Mania is considered the opposite extreme of depression. Its principal feature is euphoric behavior that lasts more than a week. Hypomania is a less severe form of mania. Bipolar disorder (BD) is characterized by an alternation of several depressive and maniacal episodes. It affects about 0.5–1.5% of the general population. Manic episodes are more frequent in men. In BD, psychotic symptoms are more frequent than in isolated depression [24]. The association between BD and HS has been evaluated in the literature. Bitan et al. [25] found a significantly higher prevalence of BP in HS than in controls (0.7% vs. 0.1%, with OR 4.7, *p* < 0.001). Moreover, we have to consider that patients with BD are usually treated with lithium. Lithium is a tough handling drug. It is well known that this drug is arrhythmogenic, thyrotoxic and nephrotoxic. Many cutaneous disorders induced by lithium have been reported. Psoriasis is the most reported, but some case series mention lithium-induced HS. [26]. The mechanisms of HS induction are not well understood but are supposed to be similar to those of lithium-induced psoriasis. Lithium inhibits adenyl cyclase and inositol monophosphate; this leads to decreased intracellular levels of cyclic adenosine monophosphate (cAMP) and inositol. The result is an increased neutrophil cell infiltration and keratinocyte proliferation, followed by follicular occlusion and inflammation, key steps in the pathogenesis of HS [26]. Some hypotheses have been suggested about the association between HS and BD. Some cases probably have been induced by lithium intake. Some authors have suggested a role of the immune system. Hidradenitis suppurativa is associated with several cytokine expressions, in particular TNFα, but also interleukin (IL) 1β, IL-17 and 10. In BD, some studies have reported increased levels of TNFα, IL-4, IL1β and IL-6. According to these studies, a primary immune system alteration maybe an explanation.

### 3.4. Schizophrenia

Schizophrenia is a psychotic disorder characterized by a deep alteration of thought. Patients usually have auditory delusions with delusional thoughts. Some patients experience “negative symptoms” with consequent deficits of daily life functions. The disease is usually chronic and patients have no or poor insight into their condition [27]. The prevalence of schizophrenia is estimated to be 1% worldwide. Bitan et al., in a nationwide cohort study, found that the prevalence of schizophrenia among HS is four times more common than in controls [28]. In particular, a higher prevalence was observed in the 30–49 age group (1.8% and 0.5%, respectively; OR 3.83, 95% CI 2.44–5.99, *p* < 0.001), and a fourfold major prevalence was demonstrated in the 50–69 age group (2% and 0.5%, respectively; OR 3.74, 95% CI 1.99–7.03, *p* < 0.001). Other less recent studies did not manage to demonstrate a statistically significant association between HS and schizophrenia. The underlying mechanisms are similar to those proposed for depression and BD. Immune system abnormalities even in this case are an issue of studies. The authors propose also the role of some environmental factors. Some authors believe that an environmental stressor can elicit both HS and schizophrenia in these patients [28].

### 3.5. Suicide

Suicide is the most extreme self-harm action. It is the most common cause of death in psychiatric patients, mostly in patients with major depressive and psychotic disorders. Hidradenitis suppurativa has been shown to be significantly associated with a high suicidal risk. Phan et al. have estimated that suicide is two times more common in HS patients than in controls (OR 2.08, 95% CI: 1.27–3.42, *p* = 0.004) [29]. In the majority of cases, suicide is associated with a long period of premeditation. Thus, if these patients were correctly identified, they could be saved. In a recent short report, Álvarez et al., from a total of 136 patients, found that 21% of them presented a moderate or high suicide risk [30]. Distinguishing a group with moderate–high suicidal risk versus low risk, the group with higher risk had a slightly more severe disease (mean Hurley 1.98 vs. 2.13) and a more delayed diagnosis (12.68 years vs. 13.66), while in both groups the inguinal region was the most affected. The risk was estimated to be higher in patients with overt psychiatric illnesses and in treatment with biologic drugs (OR: 2.586, 95% CI 1.044–6.409, *p* = 0.040). The authors hypothesized that the patients in treatment with biologics were those with a more advanced disease and delayed diagnosis. Although these data are controversial, pharmacovigilance registers report that patients in treatment with anti-TNFα drugs have a reduction in suicidal behavior [31]. Moreover, having a relative with HS seems to be a protective factor (OR = 0.377, 95% CI 0.150–0.951, *p* = 0.039) [20]. Usually, the gender distribution is similar between men and women, even if some studies indicate that women have a higher suicidal risk [32].

### 3.6. Substance Abuse

Drug abuse is the continuous use of a substance, even if it causes a condition of malaise. It becomes drug addiction when tolerance and withdrawal develop. Tolerance is the progressive reduction of the effect of a drug on the body with the administration of the same dose. Withdrawal syndrome comprises clinical symptoms such as insomnia, irritability, aches and pains, fatigue, tachycardia, sweating that occurs when drugs are interrupted. Garg et al. estimated that drug abuse among patients with HS was twice that in controls (4.0% vs. 2.0%), with alcohol, cannabis and opioids being the most abused substances [33]. Some authors hypothesized that pain and concomitant psychiatric disorders are the main factors involved in drug abuse among HS patients [29]. Some authors emphasize that alcohol is the most common abused substance in HS: liquor and spirits are more commonly consumed than wine, probably due to the lower socio-economic status of the patients with HS [34].

Opioids are the second most common substances of abuse, but since painkillers are prescribed by physicians to manage pain in HS patients, it is difficult to estimate the exact extent of the problem [33]. Cannabis is the third most abused substance [33]. The prevalence of cannabis consumption is significant higher in patients with HS compared to those with other dermatological conditions and the general population [35]. Nevertheless, the progressive change in cannabis regulation makes it more difficult to define when the consumption is legal and when there is an illegal abuse.

Pain is maybe the key reason for all substance abuse. It affects several aspects of daily life, such as self-care, working and social relationships, with a significant reduction of quality of life. All the main abused drugs in HS reduce pain with different mechanisms. Alcohol and pain have a controversial relationship. While alcohol is well known for its analgesic properties, it can also worsen pain. If on the one hand ethanol inhibits nociceptive transmission centrally, resulting in analgesia, on the other hand alcohol withdrawal can lead to acute pain in its rebound effects, and long-term alcohol abuse may lead to alcohol neuropathy, which is responsible for chronic pain as well [36]. Opioids include morphine and all the morphine-like drugs that can interact with endogenous opioid receptors. In particular, the binding with the mu receptor, expressed on neuronal cells, results in analgesia [37]. The role of cannabinoids in analgesia is not well understood, but it is likely due to the interaction with endogenous cannabinoid receptors and their anti-inflammatory effects. For this reason, physicians, and in particular dermatologists, should be aware that pain in HS is a huge problem that can lead to drug abuse. They should also treat pain, referring their patients to pain management specialists.

### 3.7. Pathogenesis and Molecular Biomarkers of Psychiatric Comorbidities in Chronic Inflammatory Skin Diseases

The biomolecular pathways governing the relation between chronic skin inflammation and mental health disorders have been explored in several studies. Most of the studies available in the literature principally investigated psoriasis and atopic dermatitis with the most common psychiatric conditions such as depression and anxiety (Table 1).

The alteration of the hypothalamus–pituitary–adrenal (HPA) axis was one of the first analyzed. Stressful life situations, as well as dermatological disorders, can increase the level of the adrenocorticotropic hormone (ACTH). The adrenocorticotropic hormone stimulates adrenal glands to release cortisol, which has been observed to be higher in patients with depression who express psychotic symptoms. Cortisol binds with high affinity to its receptors in the brain, in particular mineral corticoid receptors (MRs) in the hippocampus and glucocorticoid receptors (GRs) in the pituitary gland and amygdala. The overstimulation of these receptors may explain psychotic symptoms in some patients [38]. Stress also increases levels of epinephrine and norepinephrine. Catecholamine receptors are associated with the pathway of cyclic adenosine monophosphate (cAMP). Some studies have reported that in psoriasis there is an association between increased levels of cAMP and psychiatric disorders [39].

Cholecalciferol, or vitamin D, is a molecule with different functions, considered to act as a hormone. It can be consumed with the diet or synthesized by the cells from cholesterol. In particular, the skin is the site for the ultraviolet activation of vitamin D. Beyond its known effects on calcium homeostasis, vitamin D has also been studied as a regulator of the immune and inflammatory responses in the CNS. Vitamin D levels have been associated both with dermatological conditions and metal health disorders [40].

Serotonin (5-HT) is another possible biochemical mediator. Inhibitors of 5-HT uptake have been prescribed for many years to treat several psychiatric disorders. 5-HT receptors are expressed not only on neurons and glial cells, but on every body cell, including immune system ones. The alteration of the 5-HT pathways may explain both psychiatric disorders and inflammatory conditions, including cutaneous diseases, but these data are controversial in the literature [41].

Serum melatonin levels have been studied as well. They are well known to play a role in mood disorders. Chronic low-grade inflammation can disrupt melatonin secretion with the alteration of the circadian rhythm. The modification of the inner clock has been associated with different psychiatric disturbances. Low levels of melatonin were found in patients with psoriasis. However, the differences of levels of melatonin in dermatologic patients with or without psychiatric comorbidities appear to be not so statistically significant [42].

Different inflammatory cytokines have been investigated recently. In particular, TNFα, IL1B, IL6 and IL17 are the most studied, in particular from the initial synthesis of the first target therapies. Various studies have shown that increased levels of TNFα, IL1β and IL 6 are detected in patients with major depression, BD, schizophrenia and other psychiatric diseases [43,44]. These reports suggest that neuroinflammation can be responsible for psychiatric disorders in inflammatory skin pathologies. High levels of pro-inflammatory cytokines, such as IL 6 and TNFα, were measured in the cerebral spinal fluid (CSF) of patients with psychiatric diseases and were correlated to microglia activity and the reduction of astrocyte and oligodendrocyte markers in brain parenchyma [45]. Microglia cells are the macrophage resident cells in the CNS. These cells can be activated by different stimuli and secrete pro inflammatory mediators, which may lead to a cascade malfunction of the other glial cells of the CNS such as astrocytes and oligodendrocytes. The reduction of astrocyte cells may compromise the integrity of the blood–brain barrier with the recruitment and infiltration of monocytes and other inflammatory factors in the brain [45]. Thus, neuroinflammation could be the key to understanding the association between chronic inflammatory conditions and psychiatric comorbidities. In addition, increased levels of IL-12, 17 and Th 17 have been observed in the blood streams of patients with major depression, psoriasis and neuroinflammatory alterations. In particular, the administration of IL 17-A, a cytokine crucial in psoriasis pathogenesis in animal models, could activate the pathway of the nuclear factor kappa-light-chain-enhancer of activated B cells (NFκB) and p38mitogen-activated protein kinase (MAPK) [46].

Some studies have focused their attention on mast cells. In an atopic dermatitis (AD) mouse model with depressive-like behavior, a correlation with the increased infiltration of the mast cell in the dermis and the activation of microglia cells with inflammation in the CNS was found. Mast cells seem to promote inflammatory responses in the brain [47].

Another field of research concerns dopaminergic pathways. In pre-clinical studies on mice with psoriasis, the abnormal modification of mRNA levels of the brain-derived neurotrophic factor (BDNF) and tropomyosin receptor kinase B (TrkB) was found in the mouse brains. These mice developed depression–anxiety-like behavior [48]. The alterations of the striatal circuits and dopaminergic pathways may also explain symptoms such as depression in patients with HS.

## 4. Discussion

The aim of this review is to present a summary of the current knowledge about psychiatric comorbidities associated with HS. Hidradenitis suppurativa is much more common than usually appreciated, and it still remains an unusual condition for those physicians who are not dermatologists. Thus, delayed diagnosis is very frequent. The long time required for a correct diagnosis and the disfiguring features of the disease may explain the low quality of life of HS patients. Life quality is also worsened by several comorbidities associated with HS, especially psychiatric ones. Depression and anxiety are very frequent conditions in the general population and also in HS, being encountered in almost 20–30% of patients. The data of their prevalence are variable, using the criteria applied to evaluate depression and anxiety. Moreover, many HS cases could be lost in epidemiological analyzes, due to the poor knowledge of the disease among physicians. This lost population probably expresses severe depressive and anxious symptoms.

Substance abuse is another common comorbidity in HS. The main substances consumed are alcohol, opioids and cannabis. Pain is probably the main reason explaining substance abuse. Although pain is frequently encountered in HS patients, pain management can be difficult and requires a specific competence for which dermatologists are often not trained. For this reason, it is recommended to refer patients to pain specialists, both to better their life quality and to reduce the abuse of pain killers, which create a burden not only for patients’ health but also for society and national health systems.

Bipolar disorder and schizophrenia, being encountered in 1% of the population, are far less frequent psychiatric problems compared to depression, anxiety and substance abuse. Nevertheless, both BD and schizophrenia are estimated to be fourfold more common in HS patients than in non-HS patients. These two major psychiatric diseases require the earliest therapies by psychiatric specialists to ward off their alienating complications. Finally, anxiety, depression and other psychiatric diseases are associated with an increased risk of suicide. Suicide risk is described in HS, also independently of psychiatric comorbidities. Astonishingly, suicidal thoughts have been referred in more than 20% of patients. Dermatologists should keep in mind that HS patients have a high suicide risk, which is usually preceded by a long premeditation period. We should remember that asking about suicide does not lead to suicidal behaviors [49].

The secondary purpose of this review is to offer a brief overview of current updates in the pathogenesis and biomarkers of psychiatric comorbidities in skin diseases. Few studies are available on HS to date. Most studies have been conducted on well-known diseases, such as psoriasis and atopic dermatitis, and thus we have to suppose that in HS similar pathways are present. The high production of inflammatory cytokines in the skin activates the microglia cells in the CNS with consequent neuroinflammation, which seems to interfere with the key functions of the other glial cells and neurons. Most studied cytokines, such as TNFα, IL 1β, IL-6 and IL-17, are already targets of biologic drugs that can inhibit a specific cytokine or its receptors. Further studies should be conducted to explore the relation between these cytokines, biologic drugs and psychiatric comorbidities. Nowadays, the only approved biologic drugs in HS are anti-TNFα antibodies. The effects of this class of antibodies on psychiatric-associated disorders are controversial. New approaches include anti-IL-17 drugs. Because of the lack of large, randomized studies, the efficacy, safety and long-term effects of anti-IL-17 drugs in HS patients with psychiatric comorbidity remain unknown.

## Figures and Tables

**Table 1:** Molecular biomarkers and associated mechanisms of psychiatric comorbidities in chronic inflammatory skin disease patients.

Biomarkers	Mechanisms
HPA	ACTH- > cortisol- > MRs and GCs in hippocampus, pituitary gland and amygdala
Catecholamine	↑ Camp
5-HT	5-HT receptors on neurons, glial cells and immune cells
Melatonin	Disruption of circadian rhythm
TNFα, IL 1, IL 6	Activation of microglial cells and neuroinflammation
IL 17 A	↑ NFκB and MAPK
BDNF and TrkB	Alterations of dopaminergic pathways

## Data Availability

Not applicable.
